# Research priorities to support the development of integrated national strategies to control skin-neglected tropical diseases

**DOI:** 10.1093/trstmh/trac086

**Published:** 2022-09-21

**Authors:** Hope Simpson, Asrat Mengiste, Jean Bosco Mbonigaba, Karsor Kollie, Motto Nganda, Laura Dean, Daniel Argaw, Gail Davey, Maya Semrau

**Affiliations:** Centre for Global Health Research, Brighton and Sussex Medical School, Brighton BN1 9PX, UK; Department of Disease Control, London School of Hygiene and Tropical Medicine, London WC1E 7HT, UK; College of Health Sciences, Addis Ababa University, 2Q92+P2W, Addis Ababa, Ethiopia; Rwanda Neglected Tropical Diseases Programme, Rwanda Biomedical Centre, Ministry of Health 23VV+3MM, Kigali, Rwanda; Non Communicable and Neglected Tropical Disease Program in the Ministry of Health and Social Welfare, 8643+F7C, Monrovia, Liberia; Capacity Research Unit, Liverpool School of Tropical Medicine, Liverpool L3 5QA, UK; Capacity Research Unit, Liverpool School of Tropical Medicine, Liverpool L3 5QA, UK; Department of Control of Neglected Tropical Diseases, World Health Organization, 1211 Geneva, Switzerland; Centre for Global Health Research, Brighton and Sussex Medical School, Brighton BN1 9PX, UK; Centre for Global Health Research, Brighton and Sussex Medical School, Brighton BN1 9PX, UK

**Keywords:** integrated interventions, mapping, mass drug administration, morbidity management and disability prevention, skin-neglected tropical diseases, social science

## Abstract

**Background:**

Skin-presenting neglected tropical diseases (skin-NTDs) impose large burdens on affected people, families and communities. The NTD Roadmap 2021–2030 presents a strategic plan to guide collaborative, multisectoral action to overcome these burdens, defining targets to control, eliminate and/or eradicate skin-NTDs by 2030. One of its targets is for 40 countries to adopt integrated skin-NTD strategies. Despite this high-level support for integration, only four countries were implementing integrated skin-NTD strategies in 2020.

**Methods:**

We hosted workshops at the 2021 annual meeting of the Coalition for Operational Research on NTDs, to discuss the operationalisation of Roadmap goals into national strategies and interventions for skin-NTD control. Speakers included NTD Programme Managers from NTD-endemic countries, technical experts and researchers of different aspects of skin-NTDs.

**Results:**

Challenges include community perceptions of interventions, demonstrating the cost-effectiveness of integrated care, availability and accessibility of community-based and primary healthcare services, the quality of data on skin-NTD morbidity and changes to operational structures required for integration. Research priorities included the identification of optimal case detection platforms, evaluation of integrated care, understanding the impacts of integration on community members and community health staff and development of point-of-care diagnostics.

**Conclusions:**

The operational research priorities are intended to support the scale-up of integrated skin-NTDs programmes.

## Introduction

The NTD Roadmap 2012–2020^1^ facilitated important progress in the control of neglected tropical diseases (NTDs), including those that affect the skin (skin-NTDs), such as Buruli ulcer (BU), cutaneous leishmaniasis (CL), leprosy, lymphatic filariasis (LF), mycetoma, onchocerciasis, podoconiosis, scabies and yaws. Nevertheless, skin-NTDs continue to impose large burdens on affected people, families and communities. As well as physical health impacts including pain and physical impairment, they are associated with mental health outcomes such as distress, depression and anxiety. They can lead to detrimental social and economic outcomes, including stigma, discrimination and loss of livelihood. The NTD Roadmap for 2021–2030^2^ presents an important strategic plan to guide collaborative, multisectoral action to overcome these burdens, setting out ambitious targets to control, eliminate and/or eradicate skin-NTDs by 2030. The cross-cutting strategies of the current Roadmap include integration, multisectoral coordination, one health, universal health coverage (UHC) and country ownership. Integrated approaches to skin-NTD control, both through mass drug administration (MDA) and morbidity management and disability prevention (MMDP), are expected to lead to improved health outcomes, greater cost efficiency and effectiveness, as well as better programme management.^2^

Despite high-level support for integrated skin-NTD control, few countries implement integrated skin-NTD strategies. Challenges include the operationalisation of Roadmap goals into national strategies and interventions and the translation of global targets into country-level indicators. For this reason, a session entitled *How to Develop a Country Integrated Skin-NTD Strategy - Identifying and Filling the Research Gaps* was held at the 2021 annual meeting of the Coalition for Operational Research on NTDs (COR-NTD). The session aimed to identify knowledge gaps regarding the development of in-country integrated skin-NTD strategies, and operational research priorities to address these gaps. An initial ‘Research Links’ session, attended by around 80 people in June 2021, included presentations on integration of skin-NTD MDA, integration of skin-NTD MMDP, mapping disease distributions and overlaps, as well as perceptions and responses to skin-NTD programmes identified through applied social sciences research. These were followed by group discussions on the knowledge gaps and challenges for the development of country-integrated skin-NTD strategies, and ways to address these areas. In November 2021, a live discussion with 83 participants was held, in which the knowledge gaps and priorities were shared and ranked by participants using EasyRetro.^3^ Here, we summarise the most important outcomes emerging from those meetings, leading us to recommend research questions for operationalisation to support the development of national integrated skin-NTD strategies. This report is aimed primarily towards those working on operational issues of NTD control and management, namely, researchers, clinicians, NTD programme staff developing national control strategies and their partners and funders.

## Integration of skin-NTD MDA

**Figure 1.**
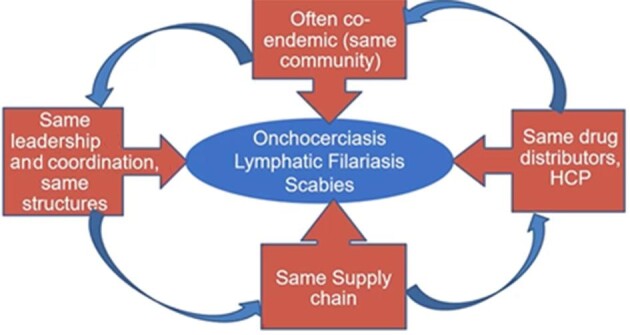
Rationale for integrating MDA for onchocerciasis, LF and scabies, building on integrated schitosomiasis and STH MDA, as implemented in Rwanda. HCP, healthcare practitioners.

Traditionally, NTD control programmes have been delivered vertically, coordinated by alliances such as the Onchocerciasis Control Programme and the Global Programme to Eliminate LF.^4,5^ Vertical structures enabled a rapid scale-up of MDA, which came to form a major component of their activities. Being managed from the top down, MDA provides a scalable platform that can be integrated across diseases, by coordinating logistics, supply chains and delivery mechanisms. Integration has enabled cost-sharing between vertical programmes, which has made MDA one of the ‘best buys’ for global health, and has provided useful lessons for integration at other levels.^6^ For several years, medicines delivered through MDA have been recognised to have spill-over benefits on the burden of other skin diseases, particularly scabies and yaws,^7,8^ and these diseases have recently been targeted respectively for control and eradication through MDA. In Rwanda, MDA against schistosomiasis and soil-transmitted helminths (STH) has been integrated for several years. Distribution is carried out through community health workers (CHWs) and schoolteachers, supported by village leaders. Data are reported from schools and villages to health centres, where they are integrated into the Health Management Information System. Building on this success, the Ministry of Health now integrates control activities for onchocerciasis, LF and scabies (Figure 1). More recently, surveillance activities (including active case searches) for LF, leprosy and podoconiosis have been integrated, also implemented through CHWs.

Despite demonstrable successes of integrated MDA, it has posed challenges and continues to do so. It necessitates changes in operational structures for implementing programmes and organisations, and requires previously independent organisations to collaborate. For larger countries with variations in NTD distribution, it may be difficult to create a national-level masterplan, which might be more effectively organised with sublevels. This can also be the case for island states, where populations can be separated by large sea distances, presenting other challenges as well. If there are a large number of co-endemic NTDs, integration of MDA can mean that populations have to take several drugs at once; in some cases up to nine tablets. In other cases, drug administration cannot be integrated because guidelines specify a particular dosage schedule. For instance, in districts of Ethiopia endemic for LF, onchocerciasis, schistosomiasis, STH (at high prevalence) and trachoma, the NTD programme planned to deliver one round of integrated ivermectin and albendazole MDA in the first week of March, followed by a round of azithromycin in the third week of March, integrated praziquantel and mebendazole or albendazole in the first week of October and ivermectin and albendazole in the third week of October.^9^ While dosage schedules such as these may be safe to modify, large safety studies would be required to provide the evidence required.^10^ Community perceptions of MDA can also be a challenge to MDA in general, and to integration specifically.

### Research priorities identified for integration of skin-NTD MDA

The group discussed opportunities to address these challenges, centring around social science approaches. It was considered important to understand community perceptions on integration at an early stage of planning. Community involvement and co-production, particularly involving affected-person advocates, could provide an opportunity to continue good community engagement.

## Integration of skin-NTD MMDP

Integrated MMDP is intended to enable cost-sharing, because several management approaches are common to several NTDs. For example, wound and lymphoedema management, surgery and rehabilitation, and active case search, could be delivered by the same health workers using the same health infrastructure and medical supplies.

A holistic, integrated package of physical and psychosocial care for three skin-NTDs (podoconiosis, LF and leprosy) was recently piloted through the EnDPoINT project in Ethiopia. The care package involved a range of interventions at the healthcare organisation, primary healthcare facility and community level, which were developed in partnership with key stakeholders and the local community (including affected persons) via workshops, interviews and focus group discussions. The integrated package showed high potential effectiveness, with statistically significant improvements in dermatological quality of life, self-reported disability, physical health outcomes such as number of acute attacks and leg/foot swelling, depression, stigma and discrimination and community participation.^11–13^ It was found to be acceptable to patients, health professionals and decision makers,^11^ and has since been scaled up to three districts in Ethiopia. Studies in Benin and Côte d'Ivoire have demonstrated the feasibility of integrated screening and treatment for leprosy, yaws and BU in co-endemic communities.^14,15^

Despite these examples, integrated care for skin-NTDs has progressed more slowly than integrated MDA, for numerous reasons. A key challenge is that the cost-effectiveness of MMDP interventions does not approach that of MDA, partly due to a lack of quality baseline data on the burden of skin-NTD–related morbidity. This burden includes not only the skin-NTDs themselves, but also diverse causes of associated morbidity, such as wounds and secondary skin infections, which may entail a greater burden. Organisational structures and funding can impede integration, as vertical approaches are often favoured by donors as being practically more straightforward to implement and monitor.^16^ Many skin-NTDs are difficult to diagnose, particularly outside of formal health facilities, and lack point-of-care diagnostics. The management of these diseases can also be complex, can entail long-term follow-up, and requires specialist clinical skills that are sometimes lacking at the lower levels of the health system.

In remote and resource-poor communities, patients often struggle to access health facilities due to transportation costs and/or because of mobility limitations due to their condition.^11^ Consequently, many activities are placed under the responsibility of peripheral health workers, who face competing demands, are not clinically trained and have variable levels of job security. The ability to deliver MMDP is also limited by basic infrastructure, particularly access to water, in many settings.

### Research priorities identified for integration of skin-NTD MMDP

Demonstrating the cost-effectiveness and/or cost-benefit of integrated vs disease-specific interventions was identified as a key research priority for MMDP, because stronger evidence in this area would support implementation by national health systems and help to attract funding. This would support staff training and the provision of medical supplies for MMDP. Further opportunities to widen access to care include the integration of case identification and management of scabies, common skin infections, diabetic foot and general wound care into existing integrated MMDP programmes, and increasing focus on social inclusion within MMDP activities.

## Mapping disease distributions and overlaps

Skin-NTDs are generally rare conditions, and are not spread evenly through populations. They often occur in clusters reflecting the distribution of environmental and socioeconomic risk factors, many of which are common to multiple diseases, resulting in overlapping distributions.^17^ To target MDA and MMDP to populations in need, and to identify areas that would benefit from integration, reliable, contemporary data on disease distributions are required. The type of data required differs between these two intervention types, reflecting the different levels at which they are delivered.

Decisions about MDA are based on endemicity within implementation units, typically measured through mapping surveys. These involve testing a defined number of people for infection or signs of disease, in a coordinated manner where possible.^18^ Because MDA is now recommended as a control strategy for scabies and yaws, the mapping of these diseases is a pressing challenge.^19,20^ Existing NTD surveillance platforms may enable data collection,^21^ although the extra costs associated with expanded surveys are likely to be a barrier to integration.

**Figure 2.**
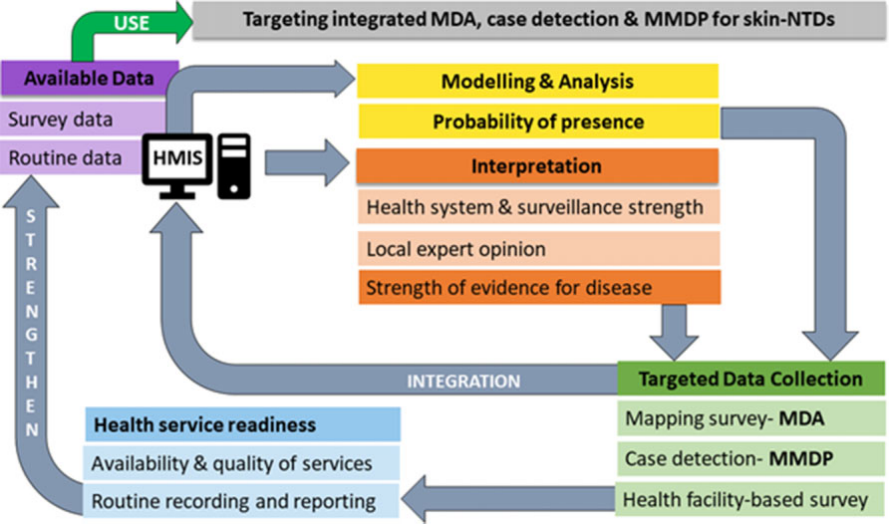
Collection, interpretation and integration of data on the distribution of skin-NTDs for the design of integrated control strategies.

The need for MMDP services is determined by the occurrence of cases at health facility level, while district-level data are required for planning and monitoring. Health facility records provide the most readily accessible (often the only available) data on skin-NTDs, but vastly underestimate disease occurrence, because patients often do not seek care through formal facilities. Routine records are typically biased due to variations in service availability, diagnostic capacity and recording, and least representative of populations with lower levels of access. This does not imply that these data should be disregarded for planning, but interpreted with reference to other information, supplemented by targeted data collection and improved for future use (Figure 2). Strengthening of recording practices and information flows has benefits beyond surveillance, for example in the monitoring of patient therapy and outcomes for diseases requiring a longer period of patient management. Active surveillance can identify unrecorded cases, although is still prone to bias, particularly if case-finders are inadequately trained or face competing demands.^22^ Disease distribution models can identify potentially endemic areas,^23–26^ which may improve the cost-effectiveness of case detection.

### Research priorities identified for understanding disease distributions and overlaps

Evaluating platforms for skin-NTD detection was considered a high priority for future research. Opportunities included community- and school-based screening, and leveraging existing platforms for the mapping of NTDs controlled primarily through preventive chemotherapy (PC-NTDs), MDA and leprosy control. The cost-effectiveness of integrated surveys was seen as a priority for evaluation, and it was suggested that costs could be further reduced if simple, specific signals—rather than prevalence estimates—could indicate the need for intervention. The comparative accuracy of diagnosis by different staff cadres was also considered an important area for investigation.

## Applied social sciences

Liberia was among the first countries to develop and implement a comprehensive, integrated strategy for NTDs requiring case management, informed by people at all levels of the health system and those affected by NTDs. Implementation of the strategy provided an opportunity to examine the feasibility and impact of integration, with social sciences approaches enabling in-depth understanding of the realities of people who the strategy would impact across the system. This showed that most participants thought the integrated approach was feasible and would help to maximise scarce resources. The benefits of integrated data flows, case finding and supervision were recognised by key informants. One national-level actor considered resistance from programmes seeking to retain their own resources as a challenge; another mentioned that the demand created by case finding could not always be met due to supply chain issues. The sustainability of programmes funded mostly by international donors was also a concern. The study also highlighted evidence gaps regarding optimal approaches for community case identification, referral and management, and requirements for capacity building and strengthening of mental health service provision.^27^ The ongoing REDRESS programme^28^ was designed to address these, using a participatory action approach with social science methodologies to inform change in four areas: early case detection and referral, human resource management, mental health and stigma reduction, and health financing. The findings are envisaged to inform the development of a national strategy for the management of severe stigmatising skin diseases.

Through the group discussion it was acknowledged that integrated programmes may face major barriers if their design does not consider the outcomes of integration for patients, communities and health staff. Settings with long-term engagement with communities and health system actors were considered to provide an opportunity to explore local aspects of feasibility, acceptability and equity, and to represent the voices of those who are typically less heard. The perspectives of CHWs and volunteers were seen as a valuable, close-to-community information source. Understanding their perspectives will help ensure that integration does not place an undue burden on them.

### Research priorities identified for applied social sciences

Participatory approaches were recommended to elucidate the priorities of different stakeholders and incorporate these into the design and scale-up of integrated programmes. Other targets for social science investigation included organisational barriers to integration, communicating the aims of integration, creating community demand and analysing patient costs. Existing and emerging evidence from such activities could be synthesised into best practice guidelines for integration.

## Conclusion

Across all areas, demonstrating the cost-effectiveness of integration over disease-specific approaches was raised as a priority to ensure programmatic sustainability. This will require the collection of detailed costing data on integrated programmes, economic data to demonstrate patient and health system costs, as well as impact measures that should take a comprehensive view of NTD burden, including mental well-being, stigma and quality of life as well as disability-adjusted life yearss. For some skin-NTD control activities, cost-effectiveness may be difficult to demonstrate because treatment can be costly and involve a long follow-up. However, there are other strong arguments for investment in skin-NTD control, especially through the primary health system, which should be advocated by the NTD community. Leprosy programmes have a successful history of advancing rights-based arguments for funding: people have a right to health, and those who are disabled or marginalised have a right to inclusion. Holistic, integrated skin-NTD programmes have wider benefits on mental well-being, stigma, prevention of disability and quality of life. Supporting integrated, mainstreamed programmes naturally supports progress towards UHC. Within endemic countries, the prioritisation of integrated skin-NTD control and related disability and inclusion activities within national and local health and development budgets would also promote sustainability and country ownership, in line with the fundamental operational shift envisaged by the NTD Roadmap.

As demonstrated by successful examples such as integrated mapping, integration does not mean integrating ‘everything’, but identifying activities and interventions that can be implemented together, using the same resources. Examples of activities that could be implemented include early case detection activities (which need not be limited to NTDs), training of primary health workers and CHWs and community health education. These will require strengthening and support of community and primary health structures. Capacity-building is required, not only to strengthen in-country health and public health systems, but also to improve the ability of the NTD research community to support these systems by addressing meaningful questions, pursuing equitable research practices and providing locally useful outputs. Co-development, sharing and standardisation of implementation tools, information, education and communication materials and best practice guidelines will also facilitate wider integration of programmatic and NGO activities.

## Research priorities for the development of country-integrated skin-NTD strategies

**Figure 3.**
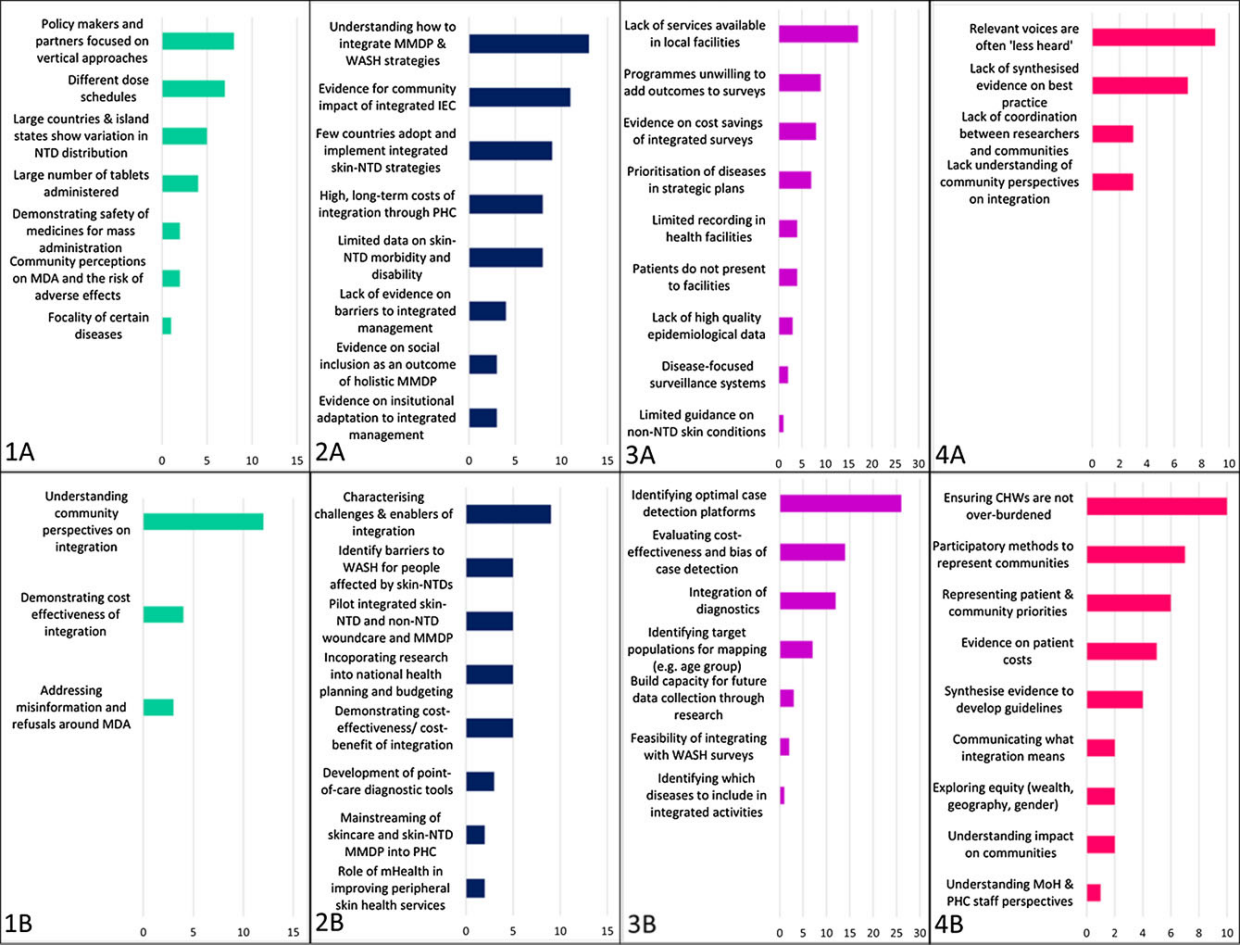
(A) Research gaps and (B) priorities to support national integrated skin-NTD strategies in (1) MMDP, (2) MDA, (3) understanding disease distributions and overlaps and (4) social sciences.

To ensure country-integrated skin-NTD strategies are impactful and well-targeted, programme managers and funders require stronger evidence on disease distributions, the effectiveness of existing and developing interventions and case detection platforms, broader representation of affected people and communities and insights from staff at all levels of the health system (Figure 3).

The identification of optimal case detection platforms is an urgent priority to improve understanding of the distribution of skin-NTD morbidity and target interventions. Existing and emerging platforms should be evaluated for cost-effectiveness, reliability, bias and equity. Inclusion of CHWs and other informal health staff in case finding and referral may increase case detection, as may the use of mobile technology, including the WHO SkinApp. Operational research in this area should have broader goals of strengthening health system and surveillance capacity through staff training and support, process evaluation and technical assistance.

The optimisation of integrated surveys to determine MDA requirements was another priority. Surveys should be optimised for cost-effectiveness, with reference to required sample sizes, target populations and survey platforms. The development of new point-of-care diagnostics would facilitate early case detection and integration across multiple diseases.

Implementation research to evaluate integrated care for skin-NTDs was a priority to facilitate integration of MMDP. Research objectives should be clearly articulated and locally relevant, including the identification of organisational barriers to integrated management, self-care challenges for patients (e.g. access to improved water, sanitation and hygiene (WASH) facilities, cost of medical supplies, quality of care received) and peripheral and clinical health worker perspectives on the feasibility of integrated care.

Understanding the impacts of integration on CHWs, to ensure that integrated activities do not overburden them and that appropriate supervision and support is in place, was a priority for applied social sciences. Innovative tools such as m-Health may be used to strengthen peripheral services.

Understanding community perspectives on integration was also a priority for MDA. Participatory research and the development of sustainable routes through which patient and community priorities can inform national and NGO programmes were seen as highly important. It was considered that some barriers to integration are not yet well characterised, and should be further investigated through organisational and implementation research. Such investigations should cover a wide range of endemic settings, because barriers to integration are expected to be diverse and context specific.

Development of new point-of-care diagnostic tools, including diagnostics that can be used for multiple NTDs.

## Data Availability

Data from the voting exercise are available on request.
